# Flow diversion versus conventional endovascular treatment for ruptured basilar artery dissecting aneurysms: a single-center cohort study

**DOI:** 10.3389/fneur.2026.1940068

**Published:** 2026-09-17

**Authors:** Bowen Yang, Ziang Chen, Jiangyu Xue, Gangqin Xu, Tongyuan Zhao, Dongyang Cai, Zhongcan Chen, Yingkun He, Yanyan He, Sijin Yang, Tianxiao Li

**Affiliations:** 1 Department of Neurosurgery, Zhengzhou University People’s Hospital, Henan Provincial People’s Hospital, Zhengzhou, Henan, China; 2 School of Computer and Artificial Intelligence, Zhengzhou University, Zhengzhou, Henan, China

**Keywords:** basilar artery, dissecting aneurysm, endovascular treatment, flow diverter, posterior circulation, subarachnoid hemorrhage

## Abstract

**Background:**

Ruptured basilar artery dissecting aneurysms (BADAs) are rare and associated with high risks of rebleeding, brainstem ischemia, and death. Evidence specific to the basilar artery remains limited, and experience derived from vertebral artery dissections may not be directly transferable because the basilar trunk supplies numerous critical pontine perforators and generally cannot be sacrificed.

**Objective:**

To describe the real-world management of a consecutive cohort of ruptured BADA and compare long-term clinical outcomes after primary flow diverter (FD)-based versus conventional endovascular treatment.

**Methods:**

This retrospective single-center cohort study included consecutive patients with angiographically confirmed ruptured BADA who underwent endovascular treatment between February 2015 and April 2024. Patients were classified according to the index endovascular treatment as the FD group or the conventional endovascular treatment group. The primary outcome was favorable functional outcome (mRS 0–2) at the last follow-up. Secondary outcomes included mortality, retreatment, complications, and descriptive radiological outcomes.

**Results:**

Twenty-two patients were included: 5 received FD-based treatment as the index strategy and 17 received conventional endovascular treatment. Fusiform morphology was more frequent in the FD group (80.0% vs. 23.5%, *p* = 0.039), while aneurysm maximum diameter was numerically larger (median 19.0 vs. 6.0 mm, *p* = 0.078). At the last follow-up, 4 of 5 patients (80.0%) in the FD group and 9 of 17 patients (52.9%) in the conventional group had a favorable outcome (*p* = 0.360). Mortality was 20.0 and 47.1%, respectively (*p* = 0.360).

**Conclusion:**

Primary FD-based reconstruction was feasible in selected ruptured BADA, particularly in anatomically complex or fusiform lesions. Given the small sample, non-randomized treatment selection, and absence of statistically significant between-group differences, these findings support individualized treatment and do not establish superiority of either strategy.

## Introduction

Basilar artery dissecting aneurysm (BADA) is an exceptionally rare but life-threatening subtype of posterior circulation aneurysm. Rupture usually presents with subarachnoid hemorrhage (SAH) and is associated with substantial risks of early rebleeding, brainstem ischemia, and death. Because most published evidence combines vertebral artery, vertebrobasilar junction, and basilar trunk lesions, the natural history and optimal treatment of ruptured BADA remain poorly defined (1–4).

The basilar artery differs importantly from the vertebral artery in both anatomy and therapeutic tolerance. In selected vertebral artery dissections, treatment decisions are often dominated by the relationship between the lesion and the posterior inferior cerebellar artery, and parent artery occlusion may be feasible when collateral circulation is adequate. In contrast, the basilar trunk gives rise to numerous critical pontine perforators and generally cannot be sacrificed without a substantial risk of devastating brainstem ischemia. Reconstructive devices placed across the basilar trunk may also cover multiple perforator origins. Consequently, experience derived predominantly from vertebral artery dissecting aneurysms cannot be directly extrapolated to BADA (5–11).

Endovascular therapy has become the principal treatment approach because the basilar trunk is deep-seated and closely related to the brainstem and perforating arteries, making microsurgical repair highly challenging. Conventional strategies, including coil embolization, stent-assisted coiling, and overlapping stents, may rapidly secure the presumed rupture site but can be limited by dissecting or fusiform morphology, incomplete exclusion of the diseased arterial segment, recurrence, and ischemic complications (5–7, 10, 11).

Flow diverters (FDs) provide a reconstructive alternative by modifying inflow, covering the dissected segment, and promoting endothelialization across the diseased arterial wall (12). However, their use in the acutely ruptured posterior circulation remains controversial because dual antiplatelet therapy, perforator compromise, in-stent thrombosis, device malapposition, and delayed aneurysm occlusion may increase hemorrhagic or ischemic risk (8, 13–16).

Dedicated series of ruptured BADA are scarce. The largest previously published disease-specific cohort identified 17 ruptured BADAs across seven institutions, while earlier single-center series generally included fewer ruptured cases or mixed ruptured and unruptured basilar dissections. Direct comparisons between FD-based reconstruction and conventional endovascular treatment are particularly limited. We therefore reviewed a consecutive, single-center cohort to characterize the real-world diagnosis, treatment, complications, and long-term outcomes of ruptured BADA and to explore differences between FD-based and conventional endovascular strategies (1–4).

## Materials and methods

### Study design and ethical approval

This was a single-center retrospective comparative cohort study of consecutive patients treated at the Cerebrovascular Interventional Center of Henan Provincial People’s Hospital between February 2015 and April 2024. The study was approved by the Ethics Committee of Henan Provincial People’s Hospital/Zhengzhou University People’s Hospital under the project titled “Post-market retrospective clinical study of flow-diverting stents for the treatment of intracranial aneurysms” (approval No. 2025-028-01). The requirement for written informed consent was waived because of the retrospective design and use of de-identified clinical data.

### Patient selection

The institutional aneurysm treatment database was retrospectively reviewed for the study period. Patients were eligible if they had: (1) basilar artery dissection confirmed by multimodal imaging, defined by at least one of the following findings: a double lumen or intimal flap on digital subtraction angiography (DSA) or MR imaging, an intramural hematoma on MR imaging, or a characteristic pearl-and-string appearance consisting of aneurysmal or fusiform dilatation contiguous with an adjacent stenotic segment on DSA; (2) spontaneous subarachnoid hemorrhage as the initial clinical presentation, with the hemorrhage attributed to the basilar lesion; (3) endovascular treatment; and (4) complete clinical data and at least one valid follow-up assessment. For lesions diagnosed mainly on the basis of pearl-and-string morphology, the diagnosis was independently reviewed by at least two experienced neurointerventionalists. Differential diagnosis incorporated the DSA appearance, relevant medical and vascular risk-factor history, serum lipid testing, and the distribution of atherosclerotic changes; selected equivocal cases underwent high-resolution vessel wall MR imaging and/or laboratory evaluation for arteritis. Infection, trauma, and iatrogenic injury were excluded on the basis of the clinical history and available laboratory and imaging findings. Patients with unruptured lesions, ischemic presentation without subarachnoid hemorrhage, no endovascular treatment, or incomplete clinical or follow-up data were excluded. Patients managed without endovascular treatment were excluded because the study was designed to evaluate and compare endovascular treatment strategies (17–19).

### Grouping strategy

Patients were categorized according to the index endovascular treatment strategy. The FD group included patients in whom an FD was used as the principal reconstructive device during the index procedure; devices included Pipeline, Tubridge, FRED, and NUVA. The conventional endovascular treatment group included patients initially treated with conventional stent-assisted coiling, coil embolization alone, overlapping non-FD stents, or parent artery occlusion when anatomically feasible. Two patients initially treated conventionally later underwent salvage FD-based retreatment for residual or recurrent disease. Because all included patients presented initially with ruptured BADA, these two patients were retained in the cohort but classified in the conventional group for the primary analysis according to their index treatment.

### Endovascular treatment

All procedures were performed under general anesthesia through a transfemoral approach. Patients had not received preoperative dual antiplatelet therapy because all aneurysms were ruptured. Tirofiban was administered intraoperatively after stent or FD deployment according to institutional practice. Systemic heparinization was used during the procedure. FD size was selected according to the proximal and distal parent artery diameters, and cone-beam CT or VasoCT was used when available to assess wall apposition. Adjunctive coiling was considered when the aneurysmal component was relatively saccular and a discrete rupture cavity could be secured to promote early thrombosis, or when a large aneurysm sac required coil support near the neck to reduce the risk of device prolapse into the sac. Conventional treatment consisted mainly of stent-assisted coiling with LVIS, Enterprise, or Solitaire stents. Coil embolization alone was considered in selected patients when early neurosurgical procedures, such as cerebrospinal fluid diversion, were anticipated and avoidance of stent-related antiplatelet therapy was clinically preferable. Postoperatively, patients who received stents or FDs were treated with aspirin and clopidogrel for 3–6 months, with FD-treated patients generally receiving dual antiplatelet therapy for at least 6 months, followed by long-term aspirin monotherapy.

### Outcomes and follow-up

The primary outcome was favorable functional outcome at last follow-up, defined as an mRS score of 0-2. Secondary outcomes included all-cause mortality, retreatment for residual or recurrent aneurysm, intraprocedural complications, early rebleeding, symptomatic ischemic stroke, hydrocephalus requiring shunting, and in-stent stenosis. Early rebleeding was defined as recurrent intracranial hemorrhage occurring within 30 days after the index endovascular procedure and confirmed by cranial CT. Clinical follow-up was obtained from outpatient records and telephone interviews. Angiographic follow-up was performed by DSA, CTA, or MRA when clinically indicated. Because imaging follow-up was incomplete and performed at nonuniform intervals, radiological outcomes were summarized descriptively among patients with available follow-up imaging.

### Statistical analysis

Continuous variables were evaluated for normality using the Shapiro–Wilk test. Normally distributed variables are presented as mean ± SD and compared using the independent-samples *t*-test. Because aneurysm maximum diameter and number of coils were non-normally distributed, they are presented as median with interquartile range (IQR) and compared using the Mann–Whitney *U* test. Other non-normally distributed or ordinal variables are presented as median with range or IQR and compared using the Mann–Whitney *U* test. Categorical variables are presented as counts and percentages and compared using Fisher’s exact test when expected cell counts were small. All tests were two-sided, and *p* < 0.05 was considered statistically significant. Analyses were performed using SPSS version 26.0 and R version 4.3.3.

## Results

### Patient characteristics

Twenty-two consecutive patients with ruptured BADA were included. There were 9 men and 13 women, and the mean age was 52.8 ± 17.9 years (range 17–77 years). All patients presented with SAH. Pretreatment Hunt-Hess grades were I-II in 12 patients (54.5%), III-IV in 7 patients (31.8%), and V in 3 patients (13.6%). According to the index treatment strategy, the FD group included 5 patients and the conventional endovascular treatment group included 17 patients. Most measured baseline clinical characteristics did not differ significantly between groups. Fusiform morphology was more frequent in the FD group than in the conventional group (80.0% vs. 23.5%, *p* = 0.039). Aneurysm maximum diameter was also numerically larger in the FD group [median 19.0 mm (IQR 12.3–19.0) vs. 6.0 mm (IQR 3.9–12.0), *p* = 0.078] (Table 1).

**Table 1:** Baseline characteristics of patients with ruptured BADA.

Variable	Overall (*n* = 22)	FD group (*n* = 5)	Conventional group (*n* = 17)	*p* value
Age, years, mean ± SD	52.8 ± 17.9	45.0 ± 20.9	56.8 ± 14.9	0.169
Male sex, *n* (%)	9 (40.9)	2 (40.0)	7 (41.2)	>0.99
Hypertension, *n* (%)	12 (54.5)	2 (40.0)	10 (58.8)	0.624
Coronary heart disease, *n* (%)	3 (13.6)	1 (20.0)	2 (11.8)	>0.99
Smoking, *n* (%)	1 (4.5)	0	1 (5.9)	>0.99
Alcohol use, *n* (%)	1 (4.5)	0	1 (5.9)	>0.99
Pretreatment mRS score, median (range)	3 (1–5)	1 (1–5)	3 (1–5)	0.834
Hunt-Hess grade, *n* (%)				0.730
I-II	12 (54.5)	2 (40.0)	10 (58.8)	
III-IV	7 (31.8)	3 (60.0)	4 (23.5)	
V	3 (13.6)	0	3 (17.6)	
Aneurysm maximum diameter, mm, median (IQR)	6.6 (4.0–13.6)	19.0 (12.3–19.0)	6.0 (3.9–12.0)	0.078
Fusiform morphology, *n* (%)	8 (36.4)	4 (80.0)	4 (23.5)	0.039
Large aneurysm (>10 mm), *n* (%)	10 (45.5)	4 (80.0)	6 (35.3)	0.135
Multiple aneurysms, *n* (%)	9 (40.9)	2 (40.0)	7 (41.2)	>0.99

Values are mean ± SD, median (range or IQR), or *n* (%). BADA, basilar artery dissecting aneurysm; FD, flow diverter; mRS, modified Rankin Scale; IQR, interquartile range.

### Treatment characteristics

In the FD group, 2 patients (40.0%) underwent FD treatment alone and 3 (60.0%) underwent FD placement combined with adjunctive coiling. In the conventional group, 15 patients (88.2%) underwent conventional stent-assisted coiling and 2 (11.8%) underwent coil embolization alone. Coil use was lower in the FD group than in the conventional group (60.0% vs. 100%, *p* = 0.043), whereas the number of coils did not differ significantly [median 3 (IQR 0–6) vs. 4 (IQR 4–6), *p* = 0.498]. One patient in the FD group developed intraprocedural in-stent thrombosis, which resolved after balloon angioplasty and tirofiban infusion without residual neurological deficit. One patient in the conventional group experienced intraprocedural extension of the dissection. Early rebleeding occurred in 1 FD-treated patient and 2 conventionally treated patients (*p* > 0.99). Both conventional-group rebleeding events were fatal, whereas the FD-treated patient underwent further treatment and survived. Symptomatic ischemic stroke occurred in 3 patients in the conventional group and in no patient in the FD group (*p* > 0.99) (Table 2).

**Table 2:** Treatment characteristics, complications, and clinical outcomes.

Variable	FD group (*n* = 5)	Conventional group (*n* = 17)	*p* value
FD alone, *n* (%)	2 (40.0)	0	–
FD plus coiling, *n* (%)	3 (60.0)	0	–
Conventional stent-assisted coiling, *n* (%)	0	15 (88.2)	–
Coiling alone, *n* (%)	0	2 (11.8)	–
Coil use, *n* (%)	3 (60.0)	17 (100)	0.043
Number of coils, median (IQR)	3 (0–6)	4 (4–6)	0.498
Any intraprocedural complication, *n* (%)	1 (20.0)	1 (5.9)	0.411
Early rebleeding, *n* (%)	1 (20.0)	2 (11.8)	>0.99
Symptomatic ischemic stroke, *n* (%)	0	3 (17.6)	>0.99
Hydrocephalus requiring shunt, *n* (%)	0	2 (11.8)	>0.99
Last-follow-up mRS score, median (range)	0 (0–6)	0 (0–6)	0.312
Favorable outcome (mRS 0–2), *n* (%)	4 (80.0)	9 (52.9)	0.360
Mortality, *n* (%)	1 (20.0)	8 (47.1)	0.360
Retreatment, *n* (%)	0	6 (35.3)	0.266
In-stent stenosis <50%, *n* (%)	0	2 (11.8)	>0.99

Values are median (range or IQR) or *n* (%), except where otherwise specified. FD, flow diverter; mRS, modified Rankin Scale; IQR, interquartile range.

### Clinical outcomes

Among patients with postdischarge clinical follow-up, the median follow-up duration was 34.5 months (range 24–62 months) in the FD group and 104.5 months (range 50–134 months) in the conventional group. Final clinical status was available for all 22 patients. Favorable outcome at last follow-up was observed in 4 of 5 patients (80.0%) in the FD group and 9 of 17 patients (52.9%) in the conventional group (*p* = 0.360). Mortality was 20.0% (1/5) in the FD group and 47.1% (8/17) in the conventional group (*p* = 0.360). The median last-follow-up mRS score was 0 (range 0–6) in both groups (*p* = 0.312). Two patients initially treated conventionally subsequently underwent salvage FD treatment for residual or recurrent aneurysm. Overall, retreatment was required in 6 patients in the conventional group and none in the primary FD group (*p* = 0.266). Mild to moderate asymptomatic in-stent stenosis (<50%) was identified in 2 patients in the conventional group and in no patient in the FD group (*p* > 0.99). Of the 9 deaths, the single death in the FD group followed severe initial SAH with persistent coma. In the conventional group, 4 deaths were related to early or late aneurysm rebleeding, 2 followed severe initial SAH, and 2 were attributed to other or indeterminate causes, including cardiorespiratory arrest without imaging-confirmed rebleeding and late cardiopulmonary failure. Given the small sample and non-random treatment selection, these descriptive differences cannot establish a causal effect of treatment choice on mortality.

### Radiological outcomes

Follow-up vascular imaging was available in 7 of 22 patients and was performed at nonuniform intervals using DSA and, in selected cases, MRA. At the last available imaging assessment, 4 patients showed complete occlusion or healing and 3 had residual aneurysm filling. Two patients initially treated conventionally underwent later FD-based retreatment for residual or recurrent disease; one subsequently demonstrated complete occlusion, whereas the other had not yet undergone post-FD vascular imaging. Because imaging follow-up was available only in a subset of patients and treatment crossover occurred, radiological outcomes were analyzed descriptively without formal between-group statistical comparison.

### Illustrative case

A patient with a ruptured mid-basilar artery dissecting aneurysm measuring approximately 19 × 15 mm and accompanied by focal proximal basilar artery stenosis underwent Tubridge flow-diverter placement with six adjunctive coils. Immediate postprocedural angiography demonstrated preservation of the basilar artery and distal branches. At 3 months, digital subtraction angiography showed progressive aneurysm occlusion with maintained parent artery patency, and axial T2-weighted magnetic resonance imaging demonstrated the treated aneurysm and reconstructed basilar artery region. Follow-up angiography at 10 months and 2 years confirmed durable aneurysm occlusion and persistent basilar artery patency (Figure 1).

**Figure 1:**
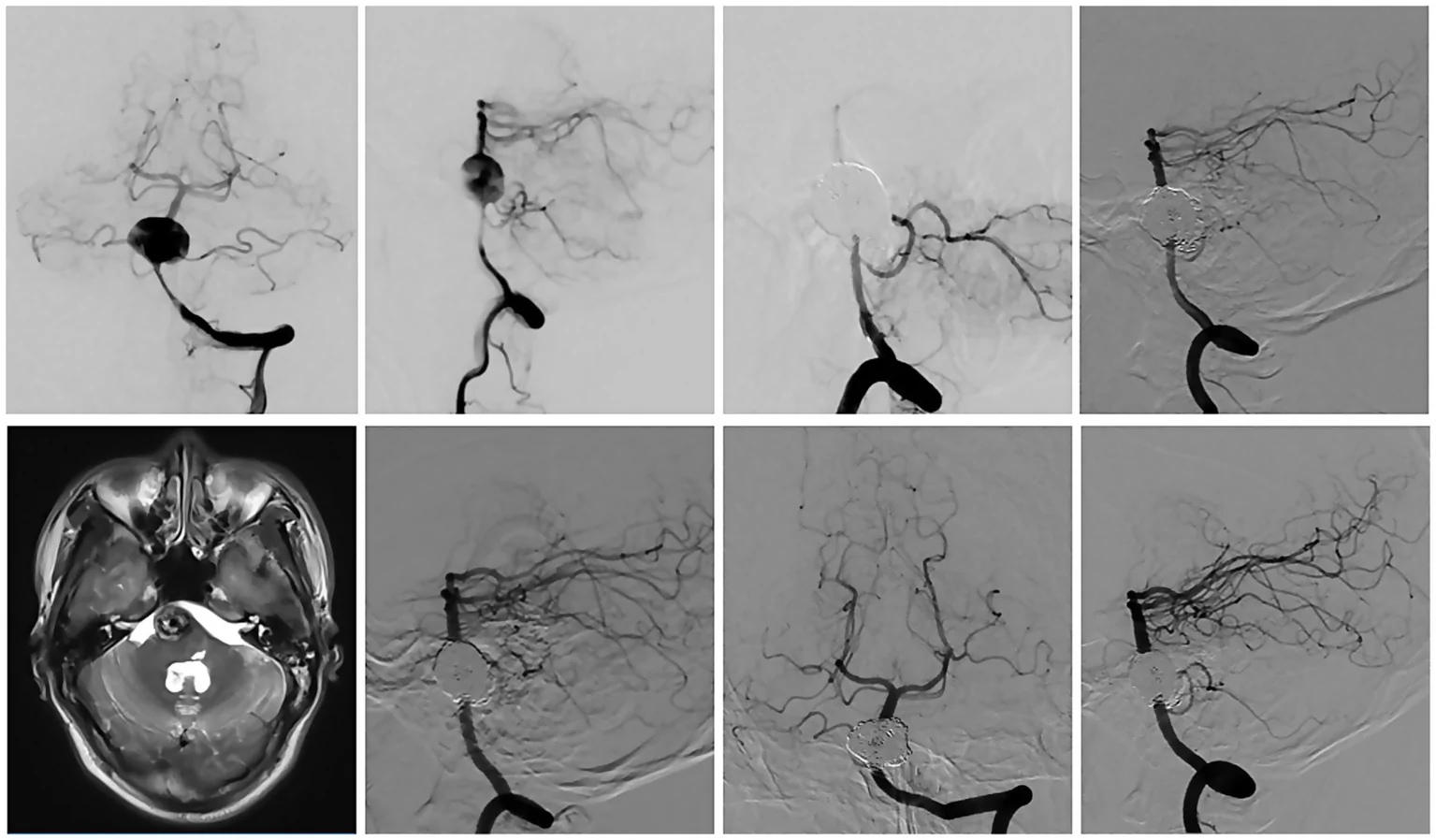
Serial imaging of a ruptured mid-basilar artery dissecting aneurysm treated with Tubridge flow-diverter-assisted coiling. **(A,B)** Preprocedural anteroposterior and lateral digital subtraction angiography images demonstrate a mid-basilar dissecting aneurysm measuring approximately 19 × 15 mm, with focal proximal basilar artery stenosis. **(C)** Immediate postprocedural angiography after deployment of a Tubridge flow diverter and six adjunctive coils shows preservation of the parent artery and distal branches. **(D)** Three-month follow-up angiography demonstrates progressive aneurysm occlusion and maintained basilar artery patency. **(E)** Axial T2-weighted magnetic resonance imaging at 3 months shows the treated aneurysm and reconstructed basilar artery region. **(F)** Ten-month follow-up angiography confirms continued aneurysm healing. **(G,H)** Two-year anteroposterior and lateral angiography demonstrates durable aneurysm occlusion and persistent basilar artery patency.

### Comparison with previous studies

Representative reports of posterior circulation, vertebrobasilar, or basilar trunk dissecting aneurysms are summarized in Table 3. Most prior endovascular cohorts pooled vertebral artery, vertebrobasilar junction, and basilar artery lesions or included unruptured aneurysms and mixed basilar trunk pathologies. Dedicated BADA studies have also included untreated patients or mixed hemorrhagic and ischemic presentations. Accordingly, direct numerical comparison should be made cautiously. The present series differs by focusing exclusively on endovascularly treated ruptured BADA, thereby providing disease-specific real-world data for a perforator-rich arterial segment whose therapeutic constraints are not equivalent to those of the vertebral artery (1–7, 20, 21, 23).

**Table 3:** Comparison with representative studies involving vertebrobasilar or basilar artery dissecting aneurysms.

Study	Cases	Ruptured proportion	Main technique	Favorable outcome	Mortality
Siddiqui 2012 (15)	7	Not specified	PED (mean 5.3 devices)	14.3%	57.1%
Natarajan 2016 (14)	12	0%	PED (mean 1.67 devices) ± coils	91.7%	0%
Guerrero 2018 (13)	9	100%	PED (1–2 devices)	77.8%	11.1%
Bi 2025 (6)	90	40%	Various EVT strategies	75.6%	8.9%
Wu 2025 (23)	20	100%	LEO/Enterprise overlapping stents + coils	89.5%	5.0%
Present study, FD group	5	100%	FD ± coils	80.0%	20.0%
Present study, conventional group	17	100%	Conventional stent-assisted coiling/coils	52.9%	47.1%
Oya 2022 (1)	17	100%	Active treatment in 7; conservative management in 10	Not reported in a directly comparable form	35.3% (overall cohort)

EVT, endovascular treatment; FD, flow diverter; PED, Pipeline Embolization Device.

## Discussion

This single-center comparative cohort study describes the real-world treatment and long-term outcomes of 22 consecutive patients with ruptured BADA. In the primary analysis based on index treatment, favorable outcome was numerically more frequent after primary FD treatment than after conventional treatment (80.0% vs. 52.9%), while mortality was numerically lower (20.0% vs. 47.1%); neither difference was statistically significant. These findings should not be interpreted as proof of superiority. Rather, they support the feasibility of FD-based reconstruction as a selective strategy in carefully chosen patients (1–4).

Although the absolute sample size was limited, to our knowledge this is one of the largest reported single-center endovascular cohorts focused exclusively on ruptured BADA. The largest previously published dedicated cohort identified 17 ruptured BADAs across seven institutions, but only a minority underwent active treatment, whereas earlier single-center studies generally included fewer ruptured cases or mixed ruptured and unruptured basilar dissections (1–4). The patients in the present study were consecutively identified from a high-volume aneurysm practice during the study period, reducing the likelihood of selective case reporting and illustrating the extreme rarity of this condition. The principal value of this study is therefore not limited to a small between-group comparison. It also provides a focused real-world account of presentation, procedural decision-making, complications, retreatment, and long-term outcome in a disease for which most published evidence consists of isolated cases or heterogeneous vertebrobasilar cohorts (5–7).

The distinction between basilar and vertebral artery dissections is clinically important. Previous studies have frequently pooled vertebral artery, vertebrobasilar junction, and basilar artery dissections, and many posterior circulation treatment series are dominated by vertebral artery lesions. Their experience may not be directly transferable to BADA. In selected vertebral artery dissections, parent artery occlusion or internal trapping can be considered when the posterior inferior cerebellar artery and collateral circulation are preserved. The basilar trunk, by contrast, supplies numerous critical pontine perforators and generally cannot be sacrificed without a substantial risk of catastrophic brainstem ischemia. Moreover, any stent or FD deployed across a basilar lesion may cover multiple perforator ostia. Thus, the tolerance of parent artery sacrifice and the perforator-related risk profile differ substantially between vertebral and basilar artery dissections, and BADA should be evaluated as a distinct therapeutic entity rather than simply as an extension of vertebral artery dissection (5–11).

The treatment of ruptured BADA remains difficult because the lesion combines the fragility of an acutely ruptured aneurysm with a dissecting process involving a perforator-rich parent artery. Unlike focal saccular aneurysms, dissecting aneurysms often lack a discrete neck and may involve a long segment of diseased arterial wall. Conventional coil embolization or stent-assisted coiling can secure the most unstable portion of the lesion, but complete exclusion may be difficult when the aneurysm is fusiform, broad-based, circumferential, or associated with a persistent false lumen (10, 11).

The potential advantage of flow diversion in BADA is mechanistically plausible. FD placement reconstructs the parent artery rather than simply filling the aneurysm cavity. By providing high metal coverage across the diseased segment, an FD reduces inflow into the dissecting aneurysm, promotes intra-aneurysmal thrombosis, supports endothelialization, and may seal the entry of the false lumen. These properties are particularly relevant for fusiform and long-segment dissections in which durable treatment requires remodeling of the pathological arterial wall. In the present study, fusiform morphology was significantly more frequent in the primary FD group, and aneurysm maximum diameter was numerically larger. This treatment-selection pattern indicates that FD was preferentially used for anatomically complex lesions and should be considered when interpreting outcome differences (5–7, 9, 12).

Conventional endovascular treatment remains important, especially in the acute ruptured setting where immediate protection of the rupture point is required and antiplatelet-related bleeding risk is a concern. Stent-assisted coiling and coil embolization alone may be faster and may immediately reduce rupture risk. However, these approaches may not fully address the underlying diseased arterial segment. Coil compaction, residual false lumen filling, and recurrence can occur when the dissection extends beyond the coil mass or when the lesion is unsuitable for dense packing. Overlapping conventional stents can increase metal coverage and provide a flow-modifying effect, but this strategy may require multiple devices and can increase procedural complexity and thromboembolic risk (10, 11, 22).

The major concern with FD treatment in ruptured posterior circulation aneurysms is safety. FD deployment requires antiplatelet therapy, which may increase hemorrhagic risk in patients requiring external ventricular drainage, shunt surgery, decompression, or other neurosurgical procedures after SAH. Basilar artery FDs also carry a risk of perforator infarction, and in-stent thrombosis is an acute concern when antiplatelet pretreatment is not feasible. In the present primary FD group, one patient developed intraprocedural in-stent thrombosis that was successfully treated without residual deficit, and one patient experienced CT-confirmed early rebleeding but survived after further treatment. These observations reinforce that FD treatment should not be applied indiscriminately; patient selection should consider rupture severity, aneurysm morphology, the presumed rupture point, perforator anatomy, the need for cerebrospinal fluid diversion, and antiplatelet feasibility (8, 13–16).

The lack of statistical significance should be interpreted in the context of disease rarity and the very small primary FD group. Although favorable outcome was numerically more frequent and mortality numerically lower after primary FD treatment, the differences were attenuated after classification by index treatment and should not be interpreted as evidence of a causal treatment effect. Mortality in the conventional group reflected heterogeneous causes, including severe initial SAH, early or late rebleeding, and other or indeterminate causes. Treatment era may also have influenced outcomes because FD use increased in later years, when devices, imaging, antiplatelet management, and operator experience had improved. Follow-up was longer in the conventional group, providing more opportunity to capture late adverse outcomes. Treatment assignment was not randomized but reflected anatomy, rupture severity, device availability, anticipated need for neurosurgical procedures, and operator judgment, introducing selection bias and confounding by indication.

Despite these limitations, the study has practical implications. First, it demonstrates that dedicated accumulation of BADA cases is possible in a high-volume center and that disease-specific analysis is preferable to pooling anatomically dissimilar vertebral and basilar lesions. Second, it supports reconstructive treatment as a feasible option in selected ruptured BADA cases while underscoring the need to preserve basilar perforators. Third, it shows that initial treatment may not definitively stabilize the diseased arterial segment and that long-term clinical and angiographic surveillance is essential. Future studies should pool multicenter BADA-specific data, standardize angiographic outcome definitions, and evaluate the interaction between morphology, rupture pattern, antiplatelet strategy, device type, and perforator-related ischemic risk (1, 5–7, 20, 21, 23).

### Limitations

This study has several limitations. First, it was retrospective, non-randomized, and conducted at a single center, which limits generalizability and introduces selection bias. Second, despite representing a comparatively large dedicated cohort for this exceptionally rare disease, the total sample and particularly the primary FD group remained small, and the study was underpowered to detect statistically significant differences. Third, the FD group was treated more frequently in the later study period, so improvements in devices, perioperative management, imaging, and operator experience may have influenced outcomes. Fourth, follow-up duration differed between groups, and the longer follow-up in the conventional group may have increased the likelihood of detecting late adverse outcomes. Fifth, angiographic follow-up was incomplete and non-uniform; consequently, radiological outcomes could be summarized only descriptively and robust between-group comparison of aneurysm occlusion, recurrence, parent artery remodeling, and in-stent stenosis was not possible. Sixth, two patients initially treated conventionally crossed over to salvage FD treatment for residual or recurrent disease. Although classification by index treatment avoids attributing their earlier survival to subsequent FD exposure, treatment crossover still complicates interpretation of long-term treatment effects. Finally, the exclusion of patients who did not undergo endovascular treatment may have introduced treatment-selection or survivor bias; therefore, the findings should not be generalized to the complete clinical spectrum of ruptured BADA.

## Conclusion

This consecutive, single-center cohort represents, to our knowledge, one of the largest reported endovascular series focused exclusively on ruptured BADA. It provides disease-specific real-world evidence for a perforator-rich and anatomically distinct condition for which treatment experience derived predominantly from vertebral artery dissections may not be directly extrapolated. Primary FD treatment was preferentially used for anatomically complex, particularly fusiform, lesions. No statistically significant difference in favorable functional outcome or mortality was observed between the index-treatment groups, and no causal superiority can be established. FD may be considered a selective reconstructive strategy for complex ruptured BADA, while treatment should remain individualized and larger multicenter BADA-specific studies are required.

## Data Availability

De-identified data may be made available from the corresponding author upon reasonable request and with appropriate institutional approval.
